# Indole Alkaloids and Phenolic Amides from the Rhizomes of *Cimicifuga heracleifolia* and Their In Vitro Soluble Epoxide Hydrolase (sEH) Inhibitory Activity

**DOI:** 10.3390/plants14121742

**Published:** 2025-06-06

**Authors:** Yanwen Sun, Chunyu Fan, Liyi Chen, Xueting Cui, Kouharu Otsuki, Mi Zhang, Feng Qiu, Liqin Ding, Wei Li

**Affiliations:** 1 Institute of Traditional Chinese Medicine, Tianjin University of Traditional Chinese Medicine, Tianjin 301617, China; 2 Tianjin Key Laboratory of Therapeutic Substance of Traditional Chinese Medicine, Tianjin University of Traditional Chinese Medicine, Tianjin 301617, Chinafengqiu20070118@163.com (F.Q.); 3 State Key Laboratory of Chinese Medicine Modernization, Tianjin University of Traditional Chinese Medicine, Tianjin 301617, China; 4 School of Chinese Materia Medica, Tianjin University of Traditional Chinese Medicine, Tianjin 301617, China; 5 Faculty of Pharmaceutical Sciences, Toho University, Funabashi 274-8510, Japan; kouharu.otsuki@phar.toho-u.ac.jp (K.O.);

**Keywords:** *Cimicifuga heracleifolia*, indole alkaloids, phenolic amides, soluble epoxide hydrolase

## Abstract

*Cimicifuga heracleifolia* is a perennial herb that belongs to the Ranunculaceae family. Its dried rhizomes are a source of Cimicifugae Rhizoma, a traditional Chinese medicine used for detoxification, the treatment of febrile diseases, and the prevention of pathogenic invasion. In the present study, a phytochemical investigation of the rhizomes of *C. heracleifolia* resulted in the isolation of three indole alkaloids (**1**–**3**) and seven phenolic amides (**4**–**10**), including three new compounds, 6-methoxy-3-(3-methyl-1-oxo-2-butenyl) 1*H* indole (**1**), (3*R*)-1-(*β*-d-glucopyranosyl)-3-hydroxy-3-(3-methylbutyl)-2-oxindole (**3**), and *N*-acetyldopamine-3-*O*-*β*-d-allopyranoside (**4**). Their structures were elucidated using extensive physicochemical and spectroscopic analyses. All the isolated compounds were evaluated for their inhibitory activity against soluble epoxide hydrolase (sEH). The results showed that cimicifugamide A (**6**) exhibited the most potent inhibitory activity, with an IC₅₀ value of 8.74 μM, followed by cimicifugamide (**7**), demethoxycimicifugamide (**8**), and *N*-*trans*-feruloyl tyramine (**10**), with IC_50_ values ranging from 15.63 to 20.58 μM. Kinetic analysis revealed that compound **6** inhibited sEH through a non-competitive mechanism.

## 1. Introduction

*Cimicifuga heracleifolia* Kom., a perennial herb belonging to the Ranunculaceae family, typically grows on grassy slopes and thickets [1]. It is distributed across north-central China, south-central China, Inner Mongolia, Korea, and the Russian Far East [2]. The dried rhizomes of this plant are a source of Cimicifugae Rhizoma, a traditional Chinese medicine that was initially recorded in the Shen Nong Ben Cao Jing (Divine Farmer’s Materia Medica) and is used for detoxification, the treatment of febrile diseases, and the prevention of pathogenic invasion [3]. Pharmacological studies on *C. heracleifolia* have revealed a wide range of biological activities, including immunomodulatory, antioxidant, antiviral, anti-osteoporotic, and anti-hyperlipidemic activities [4,5,6,7]. Previous phytochemical investigations have identified chemical constituents, including triterpenoid saponins, phenolic acids, phenolic amides, and chromones [7,8,9].

Epoxide hydrolases (EHs) constitute a small family of enzymes that play crucial roles in the detoxification of xenobiotics and the metabolic conversion of endogenous epoxides. Owing to their broad substrate specificity and diverse biological functions, EHs have attracted significant attention, particularly as therapeutic targets for the development of enzyme inhibitors [10]. Among them, soluble epoxide hydrolase (sEH), also known as EPHX2, is one of the best-characterized EHs and is widely distributed in mammalian tissues, including the liver, kidneys, and brain. The best-known substrates of sEH are the epoxyeicosatrienoic acids (EETs), which are the epoxy derivatives of arachidonic acid known for their potent anti-inflammatory properties [11,12,13]. Although the precise physiological role of human epoxide hydrolases (EHs) in cellular homeostasis remains unclear, numerous studies have demonstrated that the inhibition of sEH activity is crucial for the treatment of inflammation-related diseases, including Alzheimer’s disease, inflammatory bowel disease, arthritis, kidney injury, and pulmonary fibrosis [14,15,16].

In our previous study, we isolated enantiomeric lignans that exhibited antioxidant activity from the rhizomes of *C. heracleifolia* [17]. As part of our ongoing investigation of the chemical constituents of *C. heracleifolia*, three indole alkaloids (**1**–**3**) and seven phenolic amides (**4**–**10**), including three new compounds (**1**, **3**, and **4**), were isolated in the present study. Herein, we report on the isolation and structural elucidation of these compounds. In addition, inspired by the traditional use of *C. heracleifolia* and its resemblance to the biological effects of sEH inhibitors, as well as previous studies on the sEH inhibitory activity of structurally related indole alkaloids and phenolic amides [18,19], we evaluated the in vitro sEH inhibitory activity of compounds **1**–**10**.

## 2. Results and Discussion

### 2.1. Isolation and Structural Determination of Compounds 1−10

**Figure 1:**
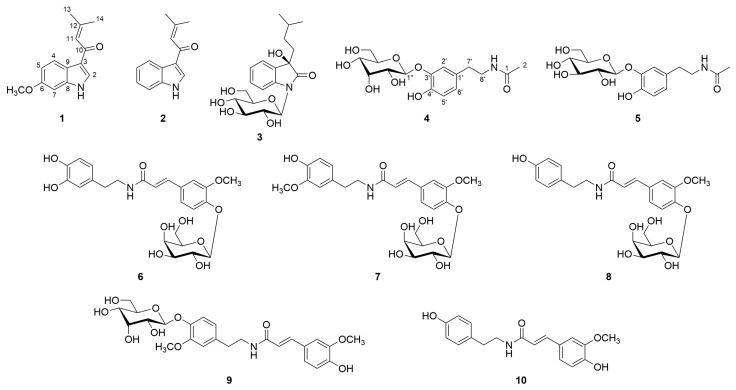
Structures of compounds **1–10** isolated from *C. heracleifolia*.

A water extract of the rhizomes of *C. heracleifolia* was separated using AB-8 macroporous adsorption resin column chromatography. Further fractionation was carried out using polyamide, Sephadex LH-20, silica gel, and ODS column chromatography, followed by reversed-phase semi-preparative HPLC, to obtain ten compounds (**1**–**10**, Figure 1), including three new compounds. The known compounds were identified as 3-(3-methyl-1-oxo-2-butenyl) 1*H* indole (**2**) [20], *N*-acetyldopamine-*3-O*-*β*-d-glucopyranoside (**5**) [21], cimicifugamide A (**6**) [22], cimicifugamide (**7**) [23], demethoxycimicifugamide (**8**) [24], cimicifugamide C (**9**) [25], and *N*-*trans*-feruloyl tyramine (**10**) [26], based on detailed NMR and MS spectroscopic analyses and by comparison with the literature data. Among them, 3-(3-methyl-1-oxo-2-butenyl) 1*H* indole (**2**) and *N*-acetyldopamine-3-*O*-*β*-d-glucopyranoside (**5**) were isolated from the genus *Cimicifuga* for the first time. Although cimicifugamide A (**6**), cimicifugamide C (**9**), and *N*-*trans*-feruloyl tyramine (**10**) have been previously reported from other *Cimicifuga* species, this is the first report of their isolation from *C. heracleifolia*.

**Figure 2:**
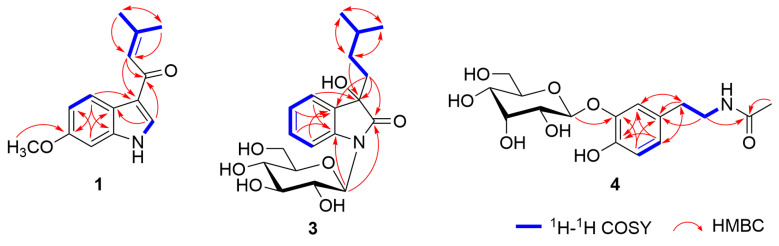
Key ^1^H–^1^H COSY and HMBC correlations of compounds **1**, **3**, and **4**.

Compound **1** was obtained as a white powder, and its molecular formula was determined to be C_14_H_15_NO_2_ based on positive-ion mode HRESIMS analysis, which exhibited an ion peak at *m*/*z* 230.1185 [M + H]^+^ (calcd for C_14_H_16_NO_2_, 230.1181). The IR absorptions revealed the presence of the NH heterocycle (3272, 1588, 1513, and 1437 cm^−1^) and carbonyl (1644 cm^−1^) moieties. In the ^1^H NMR spectrum of **1** (Table 1), the resonances at *δ*_H_ 8.20 (1H, s, H-2) and 11.77 (1H, br s, *N*-H-1) indicated the presence of a 3-substituted indole moiety [27]. Additionally, characteristic resonances were observed for a 3-methyl-1-oxo-2-butenyl moiety, including an olefinic proton at *δ*_H_ 6.80 (1H, br s, H-11) and two geminal methyl protons at *δ*_H_ 1.94 (3H, br s, H_3_-13) and 2.17 (3H, br s, H_3_-14), and a carbonyl carbon at *δ*_C_ 186.1 (C-10). A comprehensive analysis of the 1D NMR data suggested that the structure of **1** closely resembled that of the known compound 3-(3-methyl-1-oxo-2-butenyl) 1*H* indole (**2**) [20], except for the resonances arising from the benzene ring within the indole moiety. Specifically, in compound **1**, the proton resonances corresponding to a 1,3,4-trisubstituted benzene were observed at *δ*_H_ 8.12 (1H, d, *J* = 8.7 Hz, H-4), 6.80 (1H, dd, *J* = 8.7, 2.3 Hz, H-5), and 6.92 (1H, d, *J* = 2.3 Hz, H-7), along with a methoxy group at *δ*_H_ 3.77 (3H, s, 6-OCH_3_). The location of the methoxy group at C-6 was further confirmed by the HMBC correlations from H-4 to C-3 and C-6 (Figure 2). Thus, the structure of compound **1** was established as 6-methoxy-3-(3-methyl-1-oxo-2-butenyl) 1*H* indole.

Compound **3** was obtained as a brownish powder with a specific rotation of [α]^25^_D_ +28.0 (*c* 0.1, MeOH). The molecular formula was determined to be C_19_H_27_NO_7_ from the positive-ion mode HRESIMS, showing a sodium adduct ion at *m*/*z* 404.1687 [M + Na]^+^ (calcd for C_19_H_27_NO_7_Na, 404.1685). The ^1^H and ^13^C NMR spectra of **3** (Table 1) showed resonances assignable to *ortho-*substituted benzene protons at *δ*_H_ 7.32 (1H, d, *J* = 8.6 Hz, H-4), 7.08 (1H, t, *J* =9.8 Hz, H-5), 7.27 (1H, t, *J* = 9.8 Hz, H-6), and 7.14 (1H, d, *J* = 8.6 Hz, H-7); a set of protons for a *β*-glucopyranosyl moiety with its anomeric proton at *δ*_H_ 5.12 (1H, d, *J* = 9.2 Hz, Glc-H-1′); a carbonyl moiety at *δ*_C_ 177.2 (C-2); and a tertiary oxygenated carbon at *δ*_C_ 75.3 (C-3). These data indicated that **3** is a 3-substituted-3-hydroxy-2-oxindole glycoside derivative, structurally related to bruceolline N [28]. In addition, a series of proton resonances due to an isopentyl moiety were observed at *δ*_H_ 1.81 (2H, m, H_2_-10), 0.94 (2H, m, H_2_-11), 1.40 (H, m, H-12), and 0.77 (6H, d, *J* = 5.8 Hz, H_3_-13, 14). An analysis of the ^1^H−^1^H COSY and HMBC spectra revealed a spin system from H_2_-10 to H_3_-13 and H_3_-14, whereas HMBC correlations from H_2_-11 to C-3 supported the assignment of the aglycone structure as 3-hydroxy-3-isopentyl-2-oxindole (Figure 2) [29]. Compound **3** was determined to possess an *N*-glucosyl linkage, as evidenced by the anomeric carbon resonance at *δ*_C_ 81.9 (Glc*-*C-1′), which appeared significantly upfield compared to that typically observed for *O*-glucosyl groups [30]. HMBC correlations from Glc-H-1′ to C-2 and C-8 further confirmed that the *β*-glucopyranosyl moiety was attached to the N-1 of the indole moiety. Furthermore, the *β*-glucopyranosyl moiety was determined to be d-form by the HPLC analysis of the acid hydrolysate of **3** using an optical rotation detector (Figure S17).

**Figure 3:**
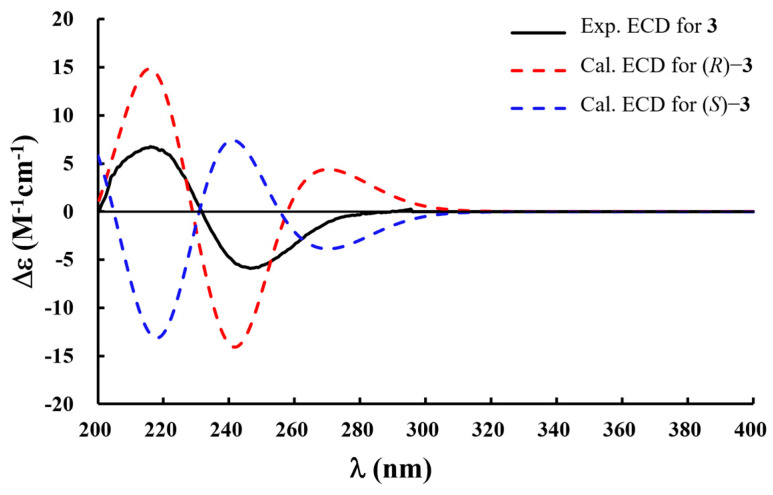
Experimental and calculated ECD spectra of compound **3**.

The absolute configuration of compound **3** was evaluated by comparing the experimental and calculated electronic circular dichroism (ECD) spectra. Conformational analysis was first conducted using the MMFF94 force field to obtain optimized geometries. Subsequently, the ECD spectra were calculated at the B3LYP/6-31+G (d) level of theory using the polarizable continuum model (PCM) in methanol. The results showed that the experimental ECD spectrum of **3** was in good agreement with the calculated spectrum of the 3*R*-isomer (Figure 3). Accordingly, the absolute configuration at C-3 was assigned as *R*. Thus, the structure of **3** was established as (3*R*)-1-(*β*-d-glucopyranosyl)-3-hydroxy-3-(3-methylbutyl)-2-oxindole.

Compound **4** was obtained as a brownish-yellow powder. Its molecular formula was determined to be C_16_H_23_NO_8_ from the negative-ion mode HRESIMS at *m*/*z* 356.1345 [M − H]^−^ (calcd for C_16_H_22_NO_8_, 356.1345). The molecular formula of **4** was identical to that of *N*-acetyldopamine-3-*O*-*β*-d-glucopyranoside (**5**), and a comprehensive analysis of the ^1^H and ^13^C NMR spectra suggested that the difference lay solely in the sugar moiety (Table 2) [21] and was considered as an epimer of **5**. Namely, the ^1^H NMR spectrum of **4** showed the presence of a dopamine moiety with proton resonances for a 1,3,4-trisubstituted benzene moiety at *δ*_H_ 6.91 (1H, d, *J* = 1.5 Hz, H-2′), 6.68 (1H, d, *J* = 8.1 Hz, H-5′), and 6.66 (1H, dd, *J* = 8.1, 1.5 Hz, H-6′); two methylene groups at *δ*_H_ 3.19 (2H, m, H_2_-8′) and 2.55 (2H, m, H_2_-7′); and an amino proton at *δ*_H_ 7.85 (1H, t, *J* = 5.5 Hz, *N*-H). In addition, the resonances corresponding to an acetyl moiety were observed at *δ*_H_ 1.77 (3H, s, H_3_-2), *δ*_C_ 169.0 (C-1), and 22.6 (C-2). For the sugar moiety, a set of proton resonances assignable to a *β*-allopyranosyl moiety were observed at *δ*_H_ 4.94 (1H, d, *J* = 7.9 Hz, H-1″), 3.45 (1H, dd, *J* = 8.2, 2.7 Hz, H-2″), 3.96 (1H, m, H-3″), 3.40 (1H, dd, *J* = 9.7, 2.7 Hz, H-4″), 3.65 (1H, m, H-5″), 3.68 (1H, m, H-6″a), and 3.48 (1H, m, H-6″b) [16]. The key HMBC correlation from All-H-1″ to *δ*_C_ 145.6 (C-3′) confirmed that the *β*-allopyranosyl moiety was attached to the C-3′ (Figure 2). Furthermore, the HPLC analysis of the acid hydrolysate of **4** using an optical rotation detector determined that the *β*-allopyranosyl moiety was in the d-form (Figure S26). Thus, the structure of **4** was established as *N*-acetyldopamine-3-*O*-*β*-d-allopyranoside.

### 2.2. Soluble Epoxide Hydrolase (sEH) Inhibitory Activity of Compounds 1−10

The sEH inhibitory activities of compounds **1**−**10** were evaluated using a fluorescence-based assay with 3-phenyl-cyano(6-methoxy-2-naphthalenyl)methyl ester-2-oxiraneacetic acid (PHOME) as the substrate [31,32]. As a result, phenolic amides **6**–**8** and **10** exhibited sEH inhibitory activity with IC_50_ values ranging from 8.74 to 20.58 μM, while indole alkaloids **1**–**3** and catecholamides **4**, **5**, and **9** did not show significant inhibitory activity (Table 3). Previous studies have demonstrated that 3-substituted-2-oxindoles inhibit sEH via a competitive mechanism [18]. The lack of inhibitory activity observed for compounds **1** and **2** was likely due to the absence of a 2-carbonyl group, which serves as a key functional group for forming a hydrogen bond with Tyr383, an essential residue at the active site of sEH [33]. Although compound **3** possessed a 2-carbonyl group, its glycosylation may sterically hinder access to the active site, thereby reducing its inhibitory activity.

**Figure 4:**
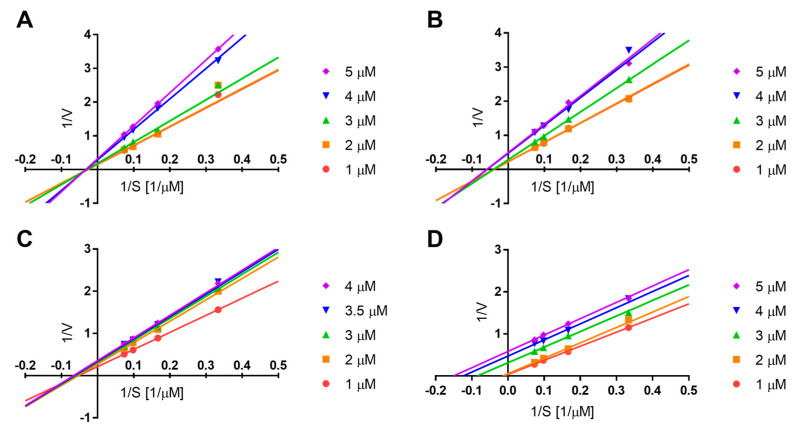
Lineweaver–Burk plots for sEH inhibition by compounds **6** (**A**), **7** (**B**), **8** (**C**), and **10** (**D**).

Among the phenolic amides **6**–**10**, which share similar aglycone scaffolds, only compound **9** showed no sEH inhibitory activity, which is suggested to result from the glycosylation of the 4-hydroxy group of the 3-methoxytyramine moiety. The comparable inhibitory activities of compounds **8** (IC_50_ = 20.58 μM) and **10** (IC_50_ = 17.16 μM) indicated that glycosylation at the 4-hydroxy group of the feruloyl moiety did not significantly affect inhibitory activity. This is supported by previous molecular docking studies showing that the 4-hydroxyl group of the amine moiety forms a hydrogen bond with the allosteric site, whereas the feruloyl moiety does not participate in binding [19]. Furthermore, the inhibition mechanisms of phenolic amides **6**–**8** and **10** were elucidated by kinetic analysis using various concentrations of compounds and the substrate, PHOME. Lineweaver–Burk plots indicated that compound **6** inhibited sEH via a non-competitive mechanism, compounds **7** and **8** via a mixed-type competitive mechanism, and compound **10** via an uncompetitive mechanism (Figure 4).

## 3. Materials and Methods

### 3.1. General Experiment Procedures

HRESIMS was performed on a Waters (Milford, MA, USA) Xevo G2-S UPLC-Q/TOF-MS. The optical rotations were measured using an AUTOPOLV polarimeter (Rudolph, Hackensack, NJ, USA). ECD spectra were measured using a JASCO J-815 spectropolarimeter (JASCO, Tokyo, Japan). NMR spectra were obtained using a 600 MHz Bruker Advance III spectrometer (Bruker, Billerica, MA, USA). Analytical HPLC was performed on a Waters e2695 system (Waters, Milford, MA, USA) equipped with a 2998 PDA detector (Waters, Milford, MA, USA). Semi-preparative HPLC was performed using a Shimadzu LC-6AD Series instrument equipped with an SPD-20A UV detector (Shimadzu, Tokyo, Japan). Column chromatography (CC) was conducted using silica gel (Qingdao Ocean Chemical Co. Ltd, Qingdao, China), octadecylsilylated (ODS) silica gel (YMC ODS-A-HG, YMC Co. Ltd., Kyoto, Japan), macroporous resin AB-8 (Tianjin Guangfu Technology Development Co. Ltd, Tianjin, China), polyamide (Changzhou Changfeng Chemical Co. Ltd, Changzhou, China), and Sephadex LH-20 (GE Healthcare, Uppsala, Sweden). sEH inhibitory activity was measured using a Spark^®^ microplate reader (TECAN, Männedorf, Switzerland). Mass spectrometry grade solvent was purchased from Sigma-Aldrich (St. Louis, MO, USA). All other reagents were of analytical and chromatographic grade (Concord Technology Co., Ltd., Tianjin, China).

### 3.2. Plant Material and sEH Information

The plant material of *C. heracleifolia* was obtained from Hebei Anguo Tongxi Chinese Herbal Decoction Pieces Co. (Anguo, China) and authenticated by Professor Lin Ma (Tianjin University of Traditional Chinese Medicine). A voucher specimen (No. 2020007) was deposited at the Institute of Traditional Chinese Medicine, Tianjin University of Traditional Chinese Medicine.

The epoxide hydrolase (sEH) enzyme is a recombinant enzyme with a concentration of 0.8 mg/mL, purchased from the Cayman Chemical Company in the United States (catalogue number: 100116, batch number: 069065) and stored at −80 °C to ensure its activity.

### 3.3. Extraction and Isolation

The dried rhizomes of *C. heracleifolia* (20.0 kg) were soaked in 10 times the amount of water for 12 h and repeatedly (×3 times) extracted for 2 h each time. The combined extract (2.8 kg) was subjected to macroporous resin AB-8 CC (130–140 Å, 4 kg, 10 × 100 cm, 12 L each), eluting with H_2_O and 40% and 95% ethanol to yield three fractions (Fr. A–C). Fr. B enriched in phenolic acids was identified by the positive reaction of ferric chloride and bromocresol green on TLC, along with the ultraviolet absorption spectrum in HPLC. Subsequently, Fr. B (248.9 g) was subjected to polyamide CC (30–60 mesh, 380 g, 8 × 60 cm), eluting with a gradient system (CH_2_Cl_2_/EtOAc = 100:0, 100:5, 100:20, 100:50, 100:80, 0:100, EtOAc/MeOH = 100:0, 100:5, 100:10, 100:30, 100:50, 100:80, 0:100, *v*/*v*, 6 L each) to afford six subfractions (Fr.B1–6). Fr.B3 (15.6 g) was separated on silica gel CC (200–300 mesh, 156 g, 4 × 60 cm), eluted by EtOAc/MeOH and EtOAc/MeOH gradiently (CH_2_Cl_2_/EtOAc = 100:0, 100:5, 100:20, 100:50, 100:80, 0:100, EtOAc/MeOH =100:0, 100:5, 100:10, 100:30, 100:50, 100:80, 0:100, *v*/*v*, 1 L each) to obtain 9 subfractions (Fr. B31–39). Fr. B37 (8.5 g) was chromatographed on silica gel CC (200–300 mesh, 85 g, 4 × 30 cm), eluted with a gradient of CH_2_Cl_2_/MeOH (100:0, 100:5, 100:20, 100:50, 100:80, 0:100, *v*/*v*, 0.8 L each) to obtain 7 subfractions (Fr. B371–377). Fr. B376 (2.1 g) was purified by Sephadex LH-20 CC (100–200 mesh, 100 g, 2.5 × 120 cm), eluting with MeOH, ODS silica gel CC (50 μm, 50 g, 6 × 40 cm) with MeOH/H_2_O and reversed-phase semi-preparative HPLC (YMC-Pack ODS-A, 4.6 × 250 mm, ODS-5 μm, YMC Co. Ltd., Kyoto, Japan) with CH_3_CN/H_2_O (10:90, *v*/*v*, 3.0 mL/min) to obtain compounds **3** (41.9 mg) and **7** (8.2 mg). Fr. B5 (68.8 g) was separated on silica gel CC (200–300 mesh, 688 g, 8 × 60 cm), eluting with CH_2_Cl_2_/EtOAc (100:0, 100:5, 100:20, 100:50, 100:80, 0:100, *v*/*v*, 6 L each) and EtOAc/MeOH (100:0, 100:5, 100:20, 100:50, 100:80, 0:100, *v*/*v*, 6 L each) to obtain 7 subfractions (Fr. B51–57). Fr. B51 (0.9 g) was chromatographed on ODS silica gel CC (50 μm, 50 g, 6 × 40 cm), eluted with a gradient of MeOH/H_2_O (40:60, 50:50, 60:40, 70:30, 90:10, 100:0, *v*/*v*, 3 L each) to obtain 6 subfractions (Fr. B511–516). Fr. B516 was purified by reversed-phase semi-preparative HPLC (YMC-Pack ODS-A, 4.6 × 250 mm, ODS-5 μm) with CH_3_CN/H_2_O (15:85, *v*/*v*, 3.0 mL/min) to obtain compounds **4** (16.2 mg) and **5** (14.8 mg). Fr. B54 (17.6 g) was purified by Sephadex LH-20 CC (100–200 mesh, 350 g, 4.5 × 120 cm), eluting by MeOH, ODS silica gel CC (50 μm, 50 g, 6 × 40 cm) with MeOH/H_2_O and reversed-phase semi-preparative HPLC (YMC-Pack ODS-A, 4.6 × 250 mm, ODS-5 μm) with CH_3_CN/H_2_O (20:80, *v*/*v*, 3.0 mL/min) to obtain compounds **6** (20.6 mg), **8** (3.1 mg), and **9** (1.4 mg). Fr. B2 (21.5 g) was separated on silica gel CC (200–300 mesh, 215 g, 8 × 60 cm) using PE/CH_2_Cl_2_ (100:0, 100:5, 100:20, 100:50, 100:80, 0:100, 0:100, *v*/*v*, 5 L each) and CH_2_Cl_2_/MeOH (100:0, 100:5, 100:20, 100:50, 100:80, 0:100, 0:100, *v*/*v*, 5 L each) as eluent to obtain 9 subfractions (Fr. B21–29). Fr. B25 (4.5 g) was purified by Sephadex LH-20 CC (100–200 mesh, 350 g, 4.5 × 120 cm), eluting with MeOH, ODS silica gel CC (50 μm, 50 g, 6 × 40 cm) with MeOH/H_2_O and reversed-phase semi-preparative HPLC (YMC-Pack ODS-A, 4.6 × 250 mm, ODS-5 μm) with CH_3_CN/H_2_O (15:85, *v*/*v*, 3.0 mL/min) to obtain compounds **1** (3.1 mg), **2** (3.2 mg), and **10** (8.1 mg).

6-Methoxy-3-(3-methyl-1-oxo-2-butenyl) 1*H* indole (**1**): White powder; UV (MeOH) *λ*_max_ (log*ε*):250 (4.27), 203 (4.14) nm; IR (KBr) *υ*_max_: 3272, 1644, 1588, 1513, 1437, 1142, 737 cm^–1^; ^1^H and ^13^C NMR spectroscopic data, see Table 1, HRTOFESIMS (positive) *m*/*z* 230.1185 [M + H]^+^ (calcd for C_14_H_16_NO_2_, 230.1181).

(3*R*)-1-(*β*-d-Glucopyranosyl)-3-hydroxy-3-(3-methylbutyl)-2-oxindole (**3**): Brownish powder; [α]^25^_D_ +28.0 (*c* 0.1, MeOH); UV (MeOH) λ_max_ (log*ε*): 246 (4.73), 209 (5.45) nm; ECD (MeOH) *λ* (Δ*ε*) 270 (+3.00), 235 (−7.00), 215 (+7.00) nm; IR (KBr) *υ*_max_: 2313, 2181, 1718, 1530, 726 cm^–1^; ^1^H and ^13^C NMR spectroscopic data, see Table 1; HRTOFESIMS (positive) *m*/*z* 404.1687 [M + Na]^+^ (calcd for C_19_H_27_NO_7_Na, 404.1685).

*N*-Acetyldopamine-3-*O*-*β*-d-allopyranoside (**4**): Brownish yellow powder; UV (MeOH) *λ*_max_ (log*ε*): 275 (3.59), 225 (3.97), 203 (4.50) nm; IR (KBr) *υ*_max_: 3329, 1707, 1392, 1062 cm^–1^; ^1^H and ^13^C NMR spectroscopic data, see Table 2; HRTOFESIMS (negative) *m*/*z* 356.1345 [M − H]^−^ (calcd for C_16_H_22_NO_8_, 356.1345).

### 3.4. Acid Hydrolysis of Compounds 3 and 4

Compounds **3** (1.0 mg) and **4** (1.0 mg) were each dissolved in 2 M HCl (2.0 mL) and heated at 80–90 °C for 1 h. After cooling, the solution was extracted twice with ethyl acetate (EtOAc). The resulting aqueous layers were analyzed by HPLC using an optical rotation detector to identify the sugar components, with d- and l-glucose or d- and l-allose as the reference standards. The HPLC conditions were as follows: column, COSMOSIL Sugar-D (4.6 mm × 250 mm, 5 μm, Nacalai Tesque, Kyoto, Japan); mobile phase, 75% CH_3_CN; flow rate, 1.0 mL/min; and detector, optical rotation detector OR-4090 (JASCO, Tokyo, Japan).

### 3.5. ECD Calculations

Conformational analysis was conducted using the MMFF94s force field using the CONFLEX 8.0 software (CONFLEX Corporation, Tokyo, Japan). All stable conformations obtained were based on the Boltzmann distribution ≥1% at the B3LYP/6-311+G (d) level in Gaussian 16 [34], and the ECD data of the conformations were calculated using the time-dependent density functional theory (TDDFT) method at the B3LYP/6-311+G (d) level with the PCM in MeOH. Finally, the calculated ECD curves were generated by the SpecDis 1.7.1 program (University of Würzburg, Würzburg, Germany) [35].

### 3.6. sEH Inhibitory Activity

The isolated compounds **1**–**10** were assayed for inhibitory activity against sEH using our previously reported method [20]. Compounds **1**–**10** were dissolved in DMSO and diluted to final concentrations from 0.1 μM to 100.0 μM. The enzymatic reactions were performed in a 96-well plate at 37 °C, where sEH hydrolyzed the fluorogenic substrate PHOME (MedChemExpress LLC., Princeton, NJ, USA) in the presence of each compound. The fluorescence signals were measured at an excitation wavelength of 330 nm and an emission wavelength of 465 nm. Wells containing the substrate alone (without the test compounds) served as negative controls. 12-(3-Adamantylureido)-dodecanoic acid (AUDA) was used as a positive control [36]. The inhibitory activity of each compound was evaluated based on the reduction in the formation of 6-methoxy-2-naphthaldehyde, a fluorescent product generated by PHOME hydrolysis.

### 3.7. sEH Kinetic Study

The inhibitory behaviour of compounds **6**–**8** and **10** against sEH was examined by analyzing kinetic changes at varying inhibitor concentrations. Each compound was tested at concentrations ranging from 1 to 5 μM, whereas the substrate PHOME was applied at concentrations ranging from 1 to 20 μM. The experimental data were fitted to the Michaelis–Menten equation to evaluate the kinetic parameters. The mode of inhibition was further determined using Lineweaver–Burk plots. Data were analyzed and plotted using GraphPad Prism 7 (GraphPad Software Inc., San Diego, CA, USA).

## 4. Conclusions

In conclusion, three indole alkaloids (**1**–**3**) and seven phenolic amides (**4**–**10**), including three new compounds (**1**, **3**, and **4**), were isolated from the rhizomes of *C. heracleifolia*. Among them, catecholamide **6** exhibited the most potent sEH inhibitory activity via a non-competitive mechanism, whereas catecholamides **7** and **8** and phenolic amide **10** demonstrated moderate inhibitory activity. These findings provide valuable insights for further research into natural sEH inhibitors.

## Figures and Tables

**Table 1:** ^1^H and ^13^C NMR spectroscopic data for compounds **1** and **3** (DMSO-*d*_6_).

No.	1					3				
	*δ*_C_, Type		*δ*_H_ (*J* in Hz)	COSY	HMBC	*δ*_C_, Type		*δ*_H_ (*J* in Hz)	COSY	HMBC
1			11.77 br s	H-2						
2	132.1	CH	8.20 s	H-1	C-9/10	177.2	C			
3	118.4	C				75.3	C			
4	122.4	CH	8.12 d (8.7)	H-5	C-3/6/8	123.6	CH	7.32 d (8.6)	H-5	C-3/8
5	111.4	CH	6.80 dd (8.7, 2.3)	H-4		122.4	CH	7.08 t (9.8)	H-4/6	C-7/9
6	156.4	C				128.7	CH	7.27 t (9.8)	H-5/7	C-4/8
7	95.0	CH	6.92 d (2.3)		C-5/9	111.6	CH	7.14 d (8.6)	H-6	C-5/9
8	137.6	C				140.7	C	–		
9	119.9	C				131.3	C	–		
10	186.1	C				35.5	CH_2_	1.81 m	H-11	C-2/3/9
11	122.3	CH	6.80 br s	H-12	C-3/10	31.5	CH_2_	0.94 m	H-10/12	C-13/14
12	151.0	C		H-11/13/14		27.7	CH	1.40 m	H-11/13/14	
13	27.3	CH_3_	1.94 br s	H-14	C-11/14	22.4 ^a^	CH_3_	0.77 d (5.8)	H-12	C-11/12/14
14	20.8	CH_3_	2.17 br s	H-13	C-11/13	22.3 ^a^	CH_3_	0.77 d (5.8)	H-12	C-11/12/13
6-OCH_3_	55.2	CH_3_	3.77 s		C-6					
Glc*-*1′						81.9	CH	5.12 d (9.2)	H-2′	C-2/8
2′						68.3	CH	3.84 m	H-1′/3′	C-4′
3′						77.2	CH	3.30 m	H-2′/4′	C-1′
4′						69.7	CH	3.26 m	H-3′/5′	C-2′/6′
5′						79.8	CH	3.32 m	H-4′/6′	C-1′/3′
6′						60.9	CH_2_	3.72 d (11.7)	H-5′/6′	C-4′
								3.50 dd (11.7, 5.6)		

^a^ interchangeable resonances.

**Table 2:** ^1^H and ^13^C NMR spectroscopic data of compound **4** (DMSO-*d*_6_).

No.	4				
	*δ*_C_, Type		*δ*_H_ (*J* in Hz)	COSY	HMBC
1	169.0	C			
2	22.6	CH_3_	1.77 s		C-1
1′	129.8	C			
2′	117.3	CH	6.91 d (1.5)		C-4′/7′
3′	145.6	C			
4′	146.0	C			
5′	115.8	CH	6.68 d (8.1)	H-6′	C-1′/3′
6′	122.8	CH	6.66 dd (8.1, 1.5)	H-5′	C-2′/4′/7′
7′	34.7	CH_2_	2.55 m	H-8′	C-1′/2′/6′
8′	40.4	CH_2_	3.19 m	H-7′/N-H	C-1/1′
*N*-H			7.85 t (5.5)	H-8′	
1″	100.6	CH	4.94 d (7.9)	H-2″	C-3′
2″	70.5	CH	3.45 dd (8.2, 2.7)	H-1″/3″	
3″	71.1	CH	3.96 m	H-2″/4″	C-5″
4″	67.1	CH	3.40 dd (9.7, 2.7)	H-3″/5″	C-6″
5″	74.9	CH	3.65 m	H-4″/6″	C-1″
6″	61.0	CH_2_	3.68 m	H-5″	C-4″
			3.48 m		

**Table 3:** sEH inhibitory activity of compounds **1**–**10**.

**Compound**	**IC_50_ (μM) ^a^**
**1**	>100
**2**	>100
**3**	>100
**4**	>100
**5**	>100
**6**	8.74 ± 0.42
**7**	15.63 ± 0.10
**8**	20.58 ± 0.89
**9**	>100
**10**	17.16 ± 0.31
AUDA ^b^	0.004 ± 0.001

^a^ results are expressed as mean ± SD (n = 3). ^b^ positive control.

## Data Availability

All new research data are presented in this contribution.

## Supplementary Materials

The following supporting information can be downloaded at: https://www.mdpi.com/article/10.3390/plants14121742/s1, Figures S1–S5: 1D and 2D NMR spectra of compound **1**; Figure S6: HRESITOFMS data of compound **1**; Figure S7: UV spectrum of compound **1**; Figure S8: IR spectrum of compound **1**; Figures S9–S13: 1D and 2D NMR spectra of compound **3**; Figure S14: HRESITOFMS data of compound **3**; Figure S15: UV spectrum of compound **3**; Figure S16: IR spectrum of compound **3**; Figure S17: Determination of the glucose configuration of compound **3**; Figures S18–S22: 1D and 2D NMR spectra of compound **4**; Figure S23: HRESITOFMS data of compound **4**; Figure S24: UV spectrum of compound **4**; Figure S25: IR spectrum of compound **4**; Figure S26: Determination of the allose configuration of compound **4**. Figure S27: Comparison of the ^1^H NMR spectrum of compounds **4** and **5**.
