# Supplementary material for: Indole Alkaloids and Phenolic Amides from the Rhizomes of Cimicifuga heracleifolia and Their In Vitro Soluble Epoxide Hydrolase (sEH) Inhibitory Activity
Source: Plants (Basel). 2025 Jun 6;14(12):1742. doi: 10.3390/plants14121742 (PMC12196358; doi:10.3390/plants14121742)
Supplement: Supplementary file 1 [file plants-14-01742-s001.zip › plants-3655707-supplementary.pdf]

Supporting Information

# Indole Alkaloids and Phenolic Amides from the rhizomes of *Cimicifuga heracleifolia* and their *In Vitro* Soluble Epoxide Hydrolase (sEH) Inhibitory Activity

Yanwen Sun [1,2,3,†], Chunyu Fan [2,3,4,†], Liyi Chen [2,3,4], Xueting Cui [2,3,4], Kouharu Otsuki [5], Mi Zhang [5], Feng Qiu [2,3,4], Liqin Ding [1,2,3,*] and Wei Li [5,*]

[1] Institute of Traditional Chinese Medicine, Tianjin University of Traditional Chinese Medicine, Tianjin 301617, China

[2] Tianjin Key Laboratory of Therapeutic Substance of Traditional Chinese Medicine, Tianjin University of Traditional Chinese Medicine, Tianjin 301617, China

[3] State Key Laboratory of Chinese Medicine Modernization, Tianjin University of Traditional Chinese Medicine, Tianjin 301617, China

[4] School of Chinese Materia Medica, Tianjin University of Traditional Chinese Medicine, Tianjin 301617, China

[5] Faculty of Pharmaceutical Sciences, Toho University, Funabashi 274-8510, Japan

[†] These authors have contributed equally to this work.

[*] Correspondence authors: ruby70303@163.com (L.D.); liwei@phar.toho-u.ac.jp (W.L.); Tel.: +86-2259596163 (L.D.); Tel.: +81-47-4721161 (W.L.)

# Contents

**Figure S1–S5**. 1D and 2D NMR spectra of compound **1** .................................................... S2

**Figure S6**. HRESITOFMS data of compound **1** .................................................... S4

**Figure S7**. UV spectrum of compound **1** .................................................... S5

**Figure S8**. IR spectrum of compound **1** .................................................... S5

**Figure S9–S13**. 1D and 2D NMR spectra of compound **3** .................................................... S6

**Figure S14**. HRESITOFMS data of compound **3** .................................................... S8

**Figure S15**. UV spectrum of compound **3** .................................................... S9

**Figure S16**. IR spectrum of compound **3** .................................................... S9

**Figure S17**. Determination of the glucose configuration of compound **3** by ORD (optical rotation detector) and HPLC .................................................... S10

**Figure S18–S22**. 1D and 2D NMR spectra of compound **4** .................................................... S11

**Figure S23**. HRESITOFMS data of compound **4** .................................................... S13

**Figure S24**. UV spectrum of compound **4** .................................................... S14

**Figure S25**. IR spectrum of compound **4** .................................................... S14

**Figure S26**. Determination of the allose configuration of compound **4** by ORD (optical rotation detector) and HPLC .................................................... S15

**Figure S27**. Comparison of the $^{1}$H NMR spectrum of compounds **4** and **5** .......................... S16

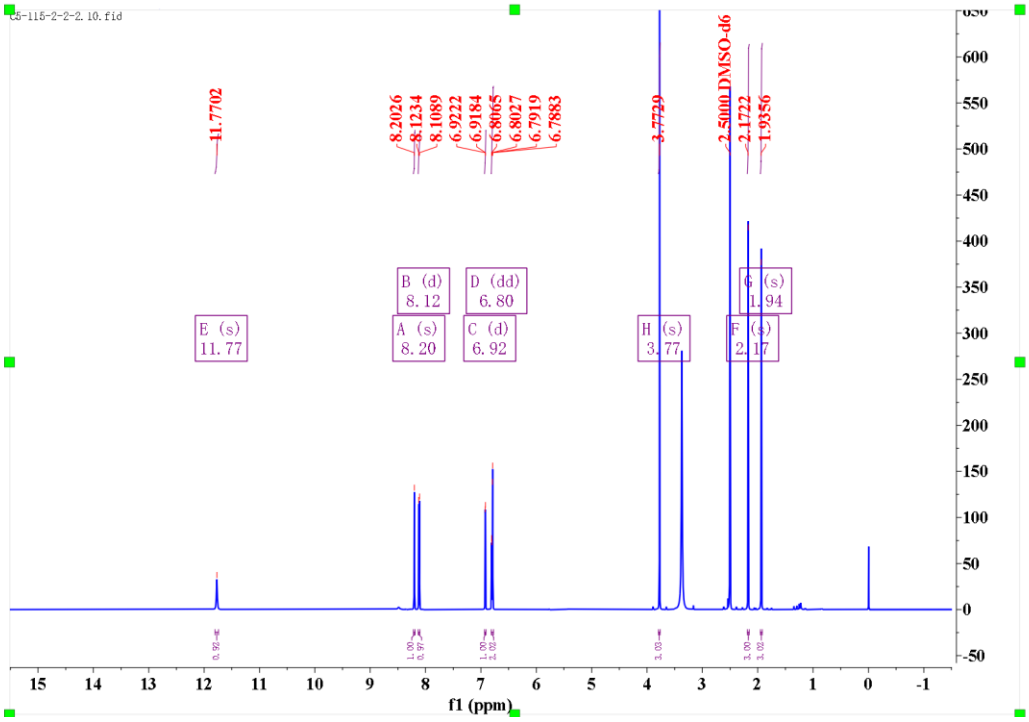


**Figure S1**. $^{1}$H NMR spectrum (600 MHz) of compound **1** (DMSO-$d_6$).

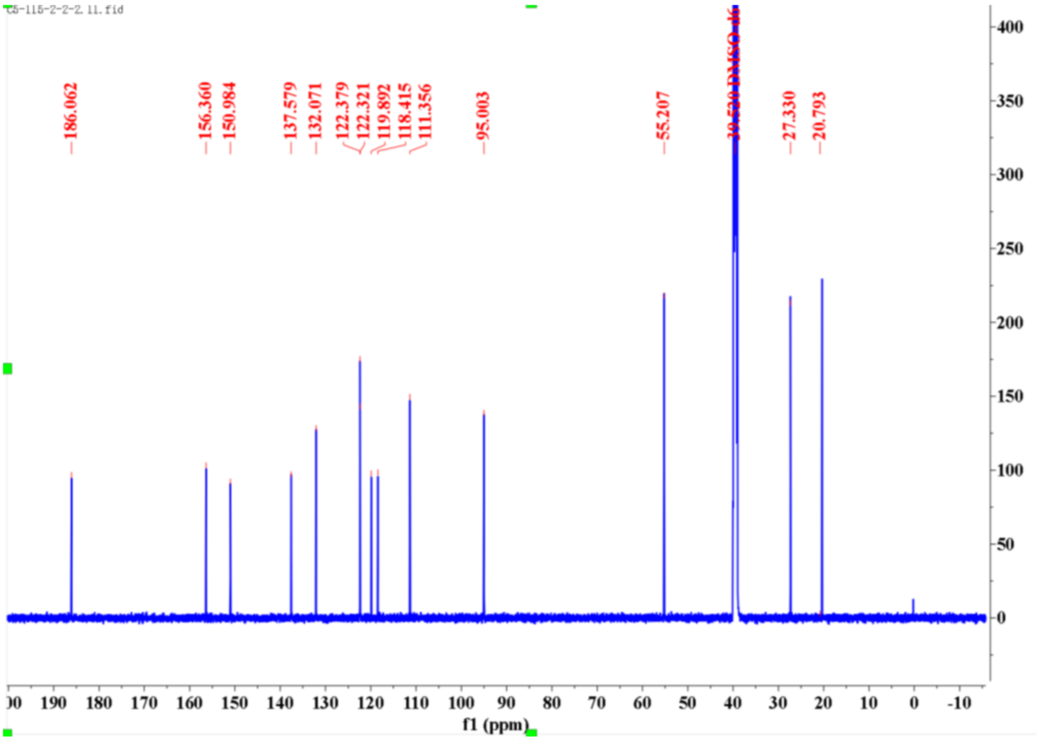


**Figure S2**. $^{13}$C NMR spectrum (150 MHz) of compound **1** (DMSO-$d_6$).

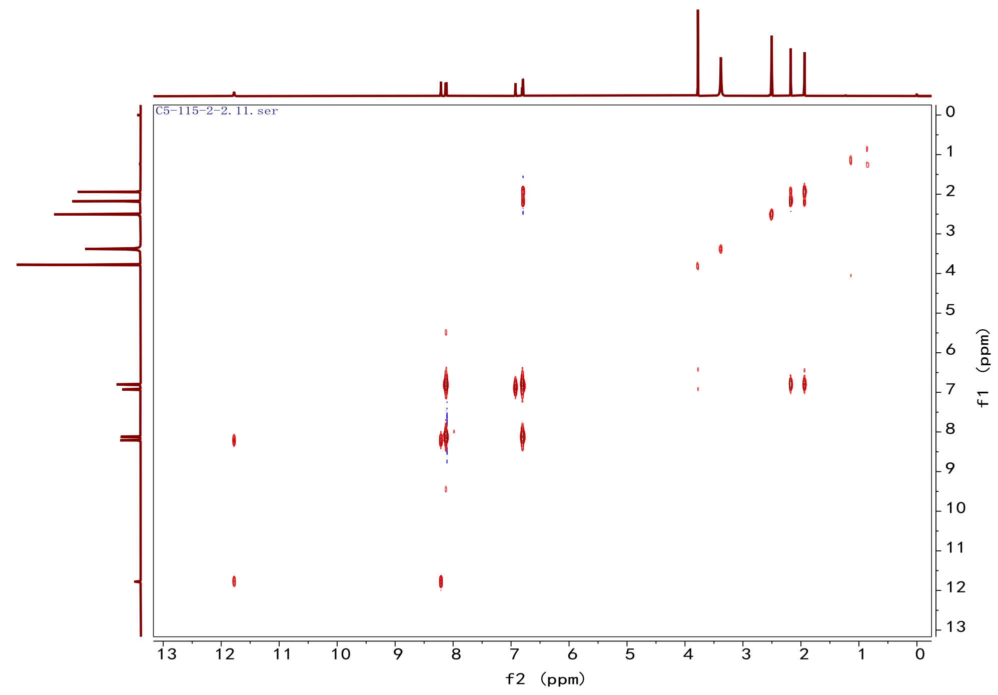


**Figure S3**. $^{1}$H-$^{1}$H COSY spectrum of compound **1** (DMSO-*d*$_6$).

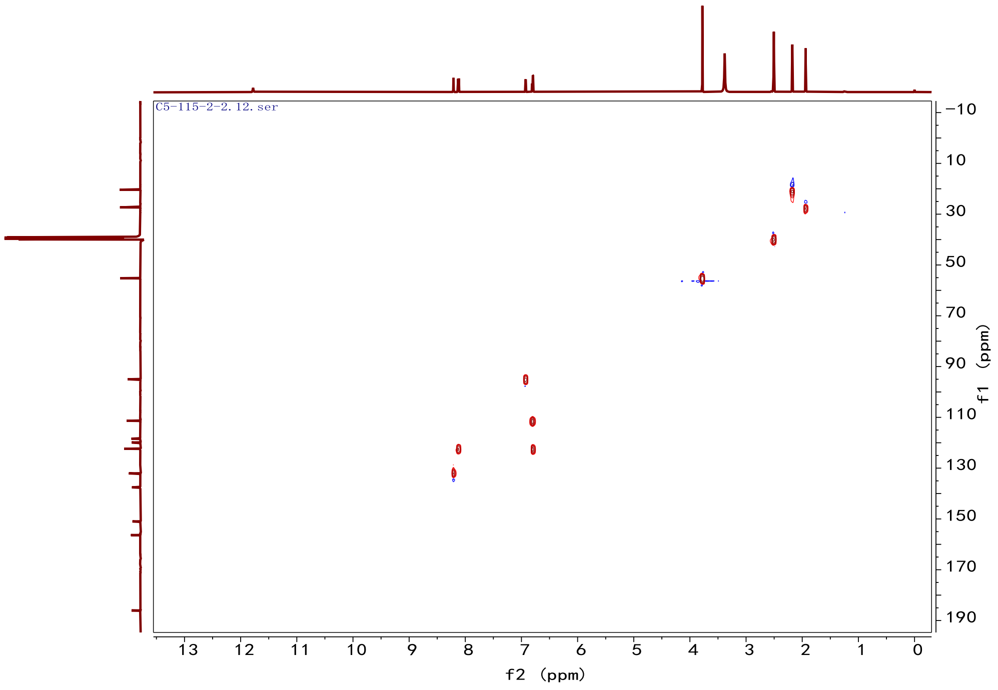


**Figure S4**. HSQC spectrum of compound **1** (DMSO-*d*$_6$).

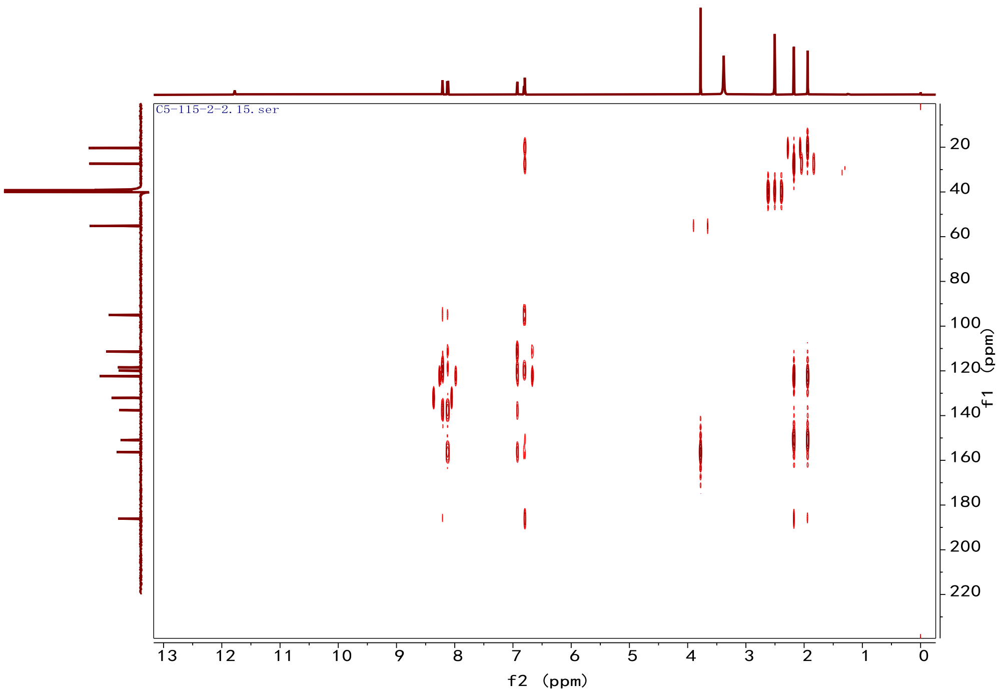


**Figure S5**. HMBC spectrum of compound **1** (DMSO-$d_6$).

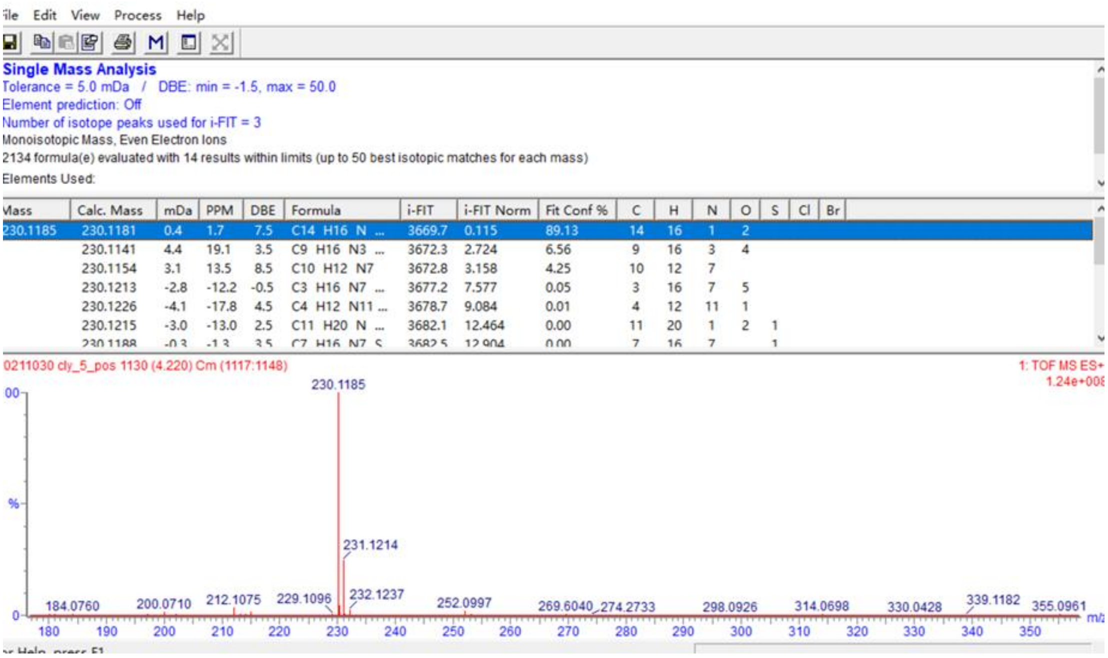


**Figure S6**. HRESITOFMS data of compound **1**.

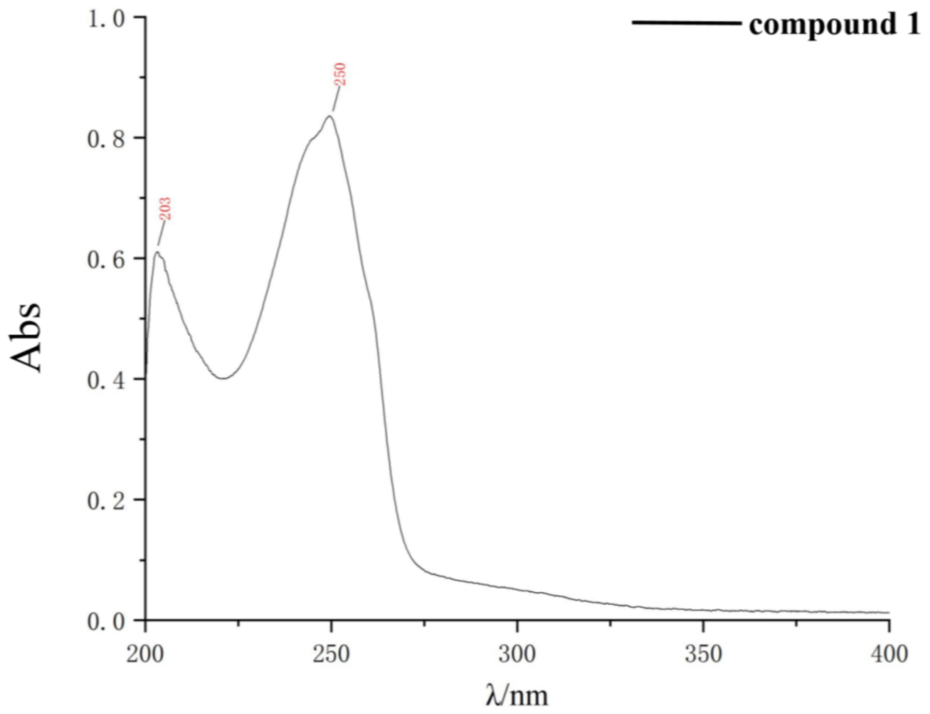


**Figure S7**. UV spectrum of compound **1**.

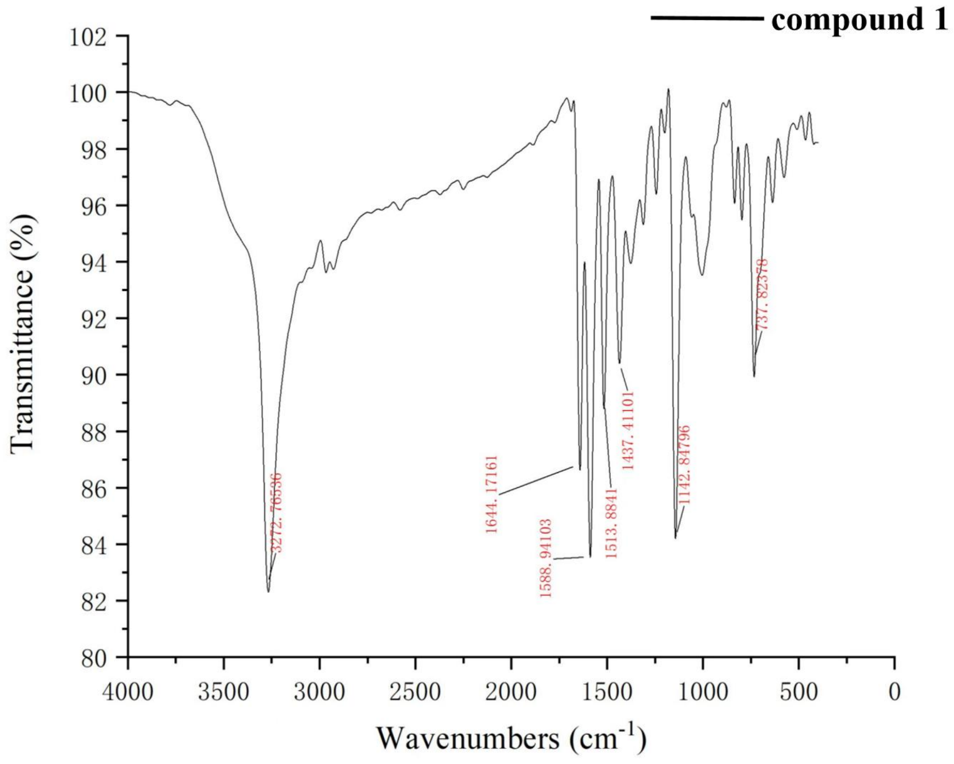


**Figure S8**. IR spectrum of compound **1**.

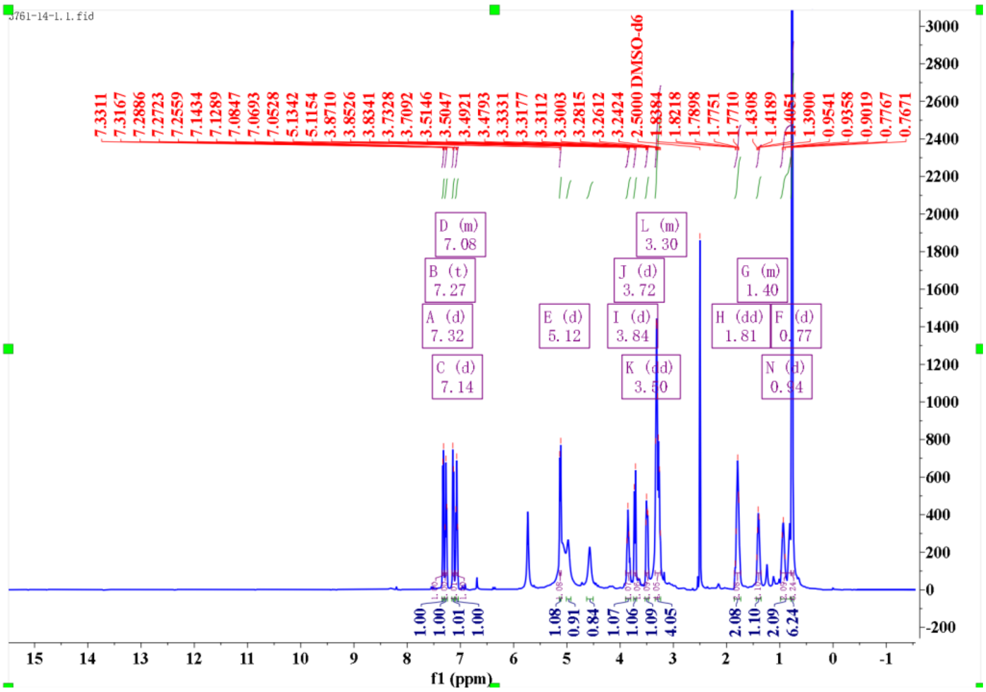


**Figure S9**. $^{1}$H NMR spectrum (600 MHz) of compound **3** (DMSO-*d*$_6$).

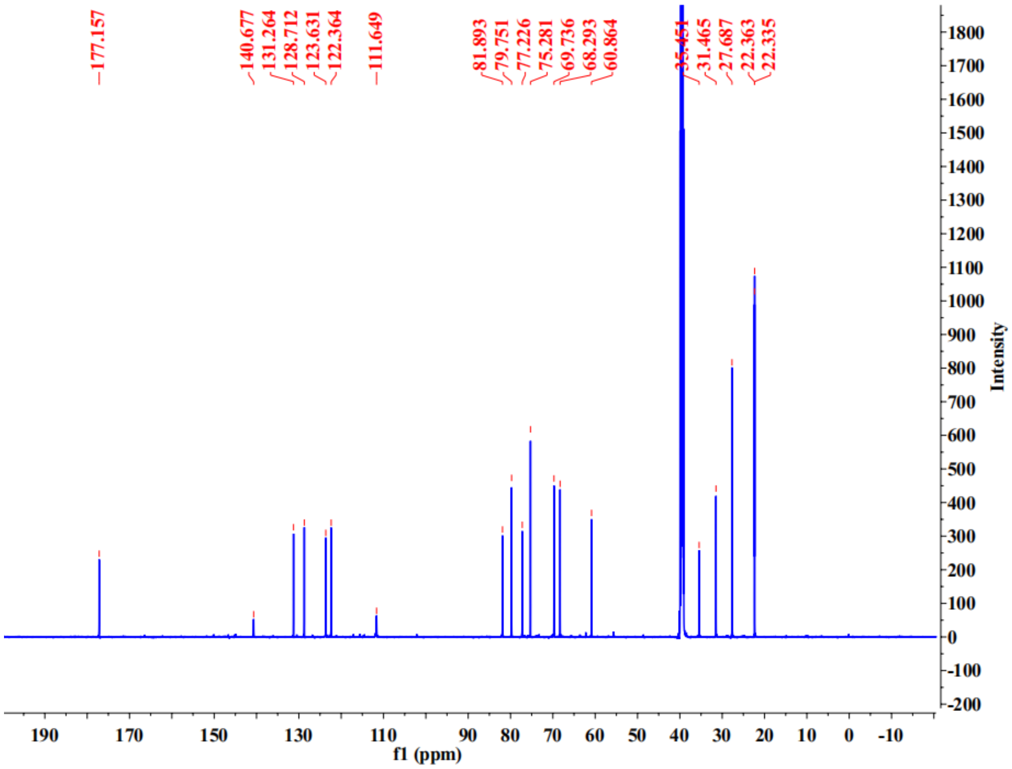


**Figure S10**. $^{13}$C NMR spectrum (150 MHz) of compound **3** (DMSO-*d*$_6$).

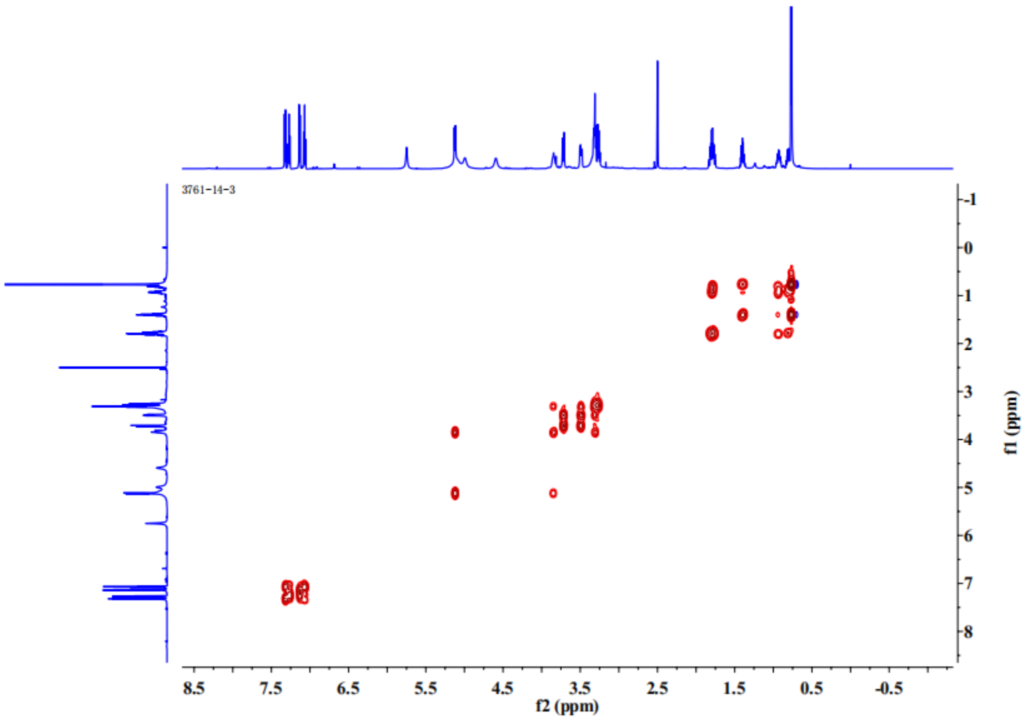


**Figure S11**. $^{1}H$-$^{1}H$ COSY spectrum of compound **3** (DMSO-*d*$_6$).

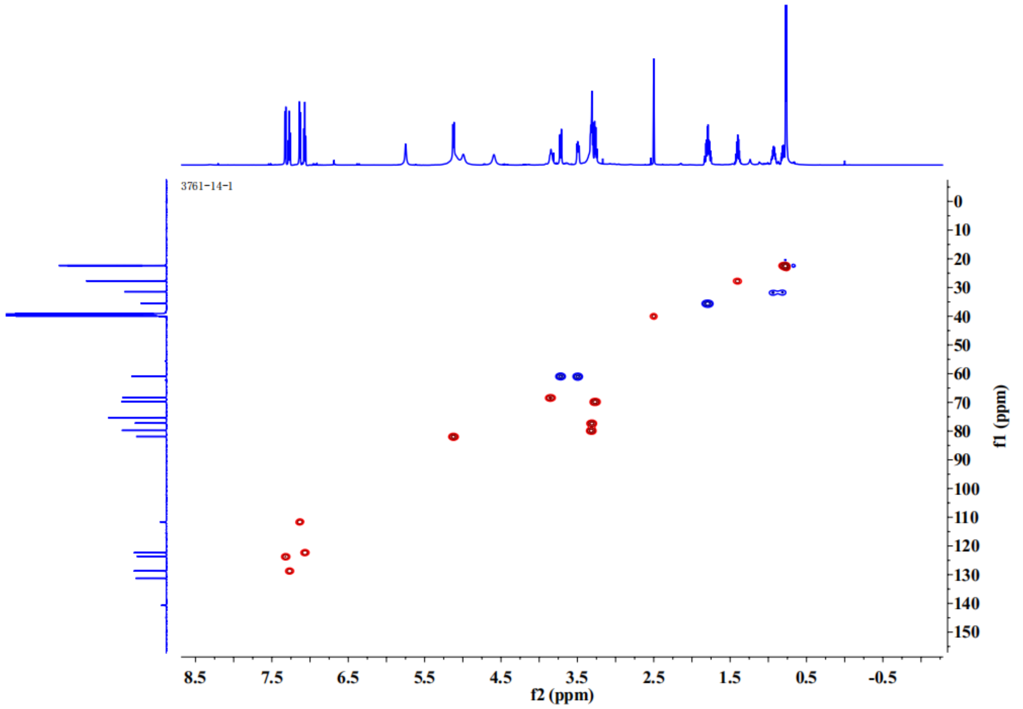


**Figure S12**. HSQC spectrum of compound **3** (DMSO-*d*$_6$).

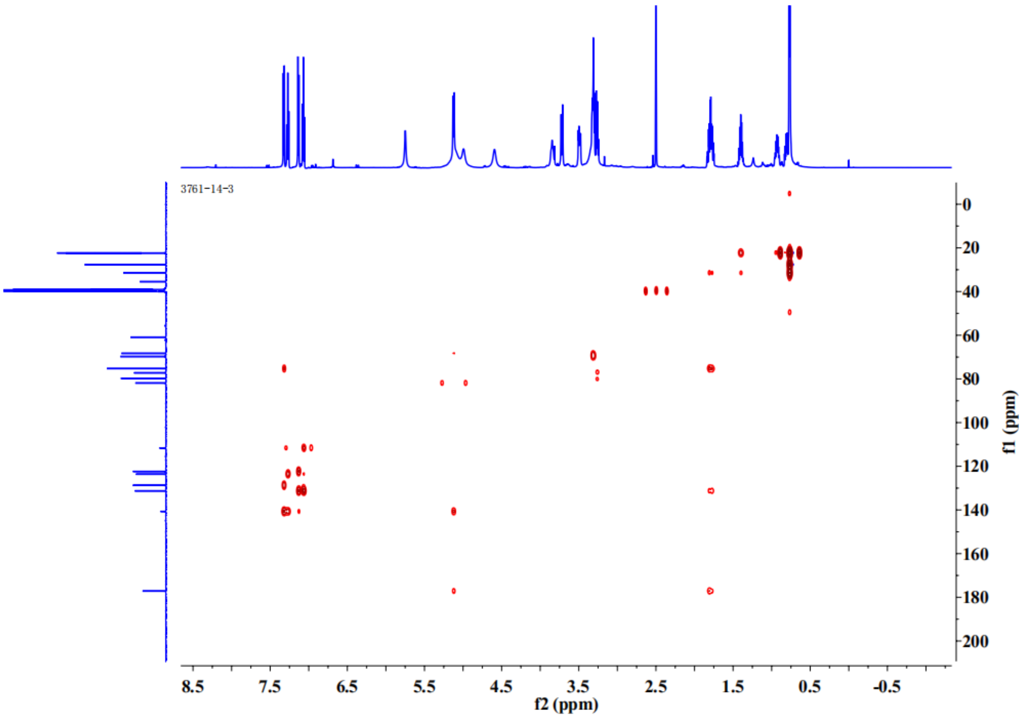


**Figure S13**. HMBC spectrum of compound **3** (DMSO-*d*6).

**Single Mass Analysis**
Tolerance = 2000.0 PPM / DBE: min = -1.5, max = 50.0
Element prediction: Off
Number of isotope peaks used for i-FIT = 3
Monoisotopic Mass, Even Electron Ions
5165 formula(e) evaluated with 2054 results within limits (up to 50 closest results for each mass)
Elements Used:

| Mass | Calc. Mass | mDa | PPM | DBE | Formula | i-FIT | i-FIT Norm | Fit Conf % | C | H | N | O | N... |
|---|---|---|---|---|---|---|---|---|---|---|---|---|---|
| 404.1687 | 404.1687 | 0.0 | 0.0 | 3.5 | C2 H18 N19 O6 | 1022.5 | 22.220 | 0.00 | 2 | 18 | 19 | 6 | |
| | 404.1688 | -0.1 | -0.2 | 0.5 | C7 H23 N13 Na5 | 1020.8 | 20.550 | 0.00 | 7 | 23 | 13 | | 5 |
| | 404.1685 | 0.2 | 0.5 | 6.5 | C19 H27 N O7 Na | 1000.3 | 0.006 | 99.38 | 19 | 27 | 1 | 7 | 1 |
| | 404.1690 | -0.3 | -0.7 | -0.5 | C4 H23 N13 O8 ... | 1020.7 | 20.429 | 0.00 | 4 | 23 | 13 | 8 | 1 |
| | 404.1691 | -0.4 | -1.0 | 9.5 | C21 H25 N3 O ... | 1011.6 | 11.324 | 0.00 | 21 | 25 | 3 | 1 | 3 |
| | 404.1682 | 0.5 | 1.2 | 10.5 | C17 H22 N7 O5 | 1005.8 | 5.550 | 0.39 | 17 | 22 | 7 | 5 | |
| | 404.1680 | 0.7 | 1.7 | 1.5 | C3 H20 N17 O4 | 1022.1 | 21.842 | 0.00 | 3 | 20 | 17 | 4 | 2 |

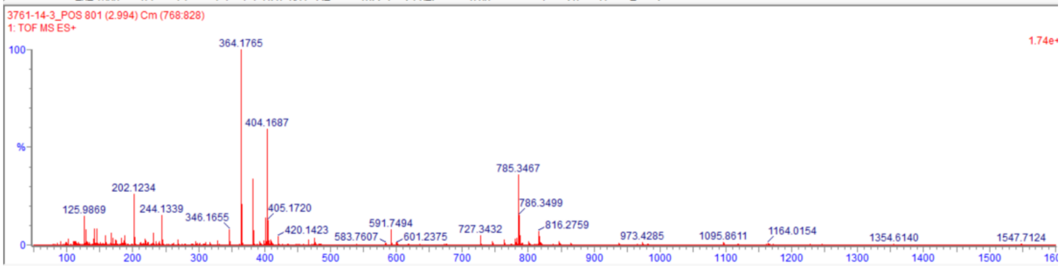


**Figure S14**. HRESITOFMS data of compound **3**.

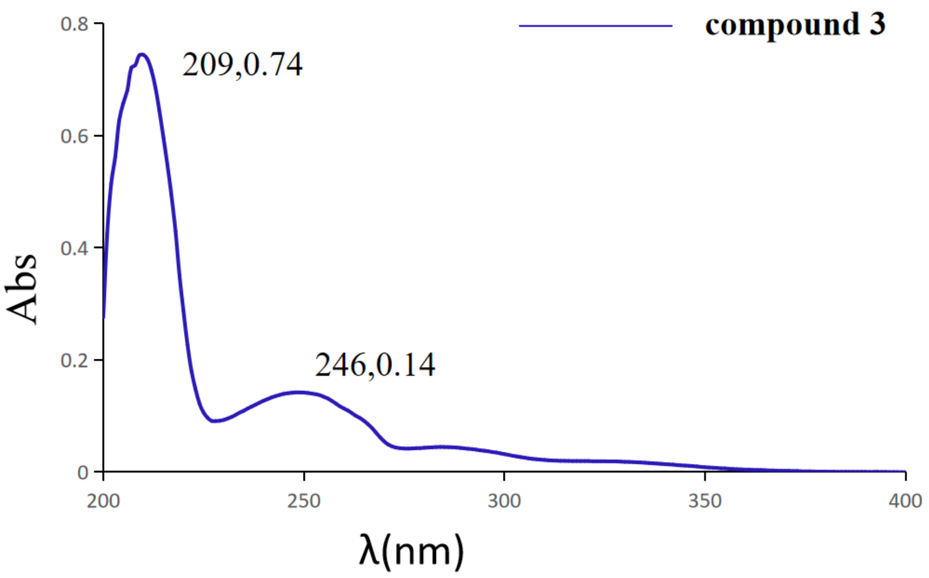


**Figure S15**. UV spectrum of compound **3**.

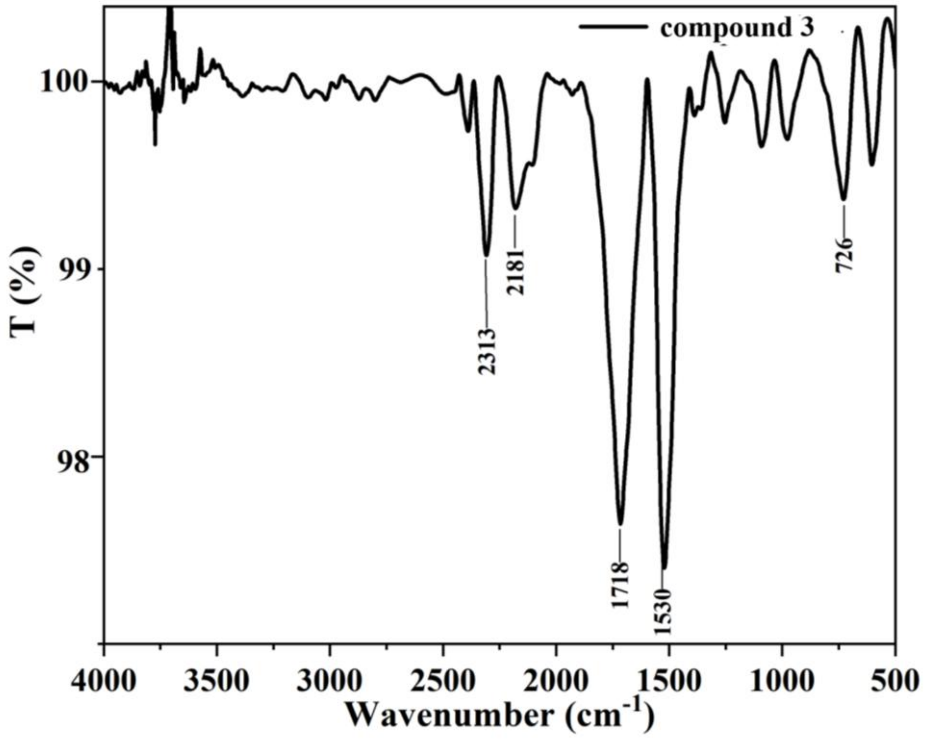


**Figure S16**. IR spectrum of compound **3**.

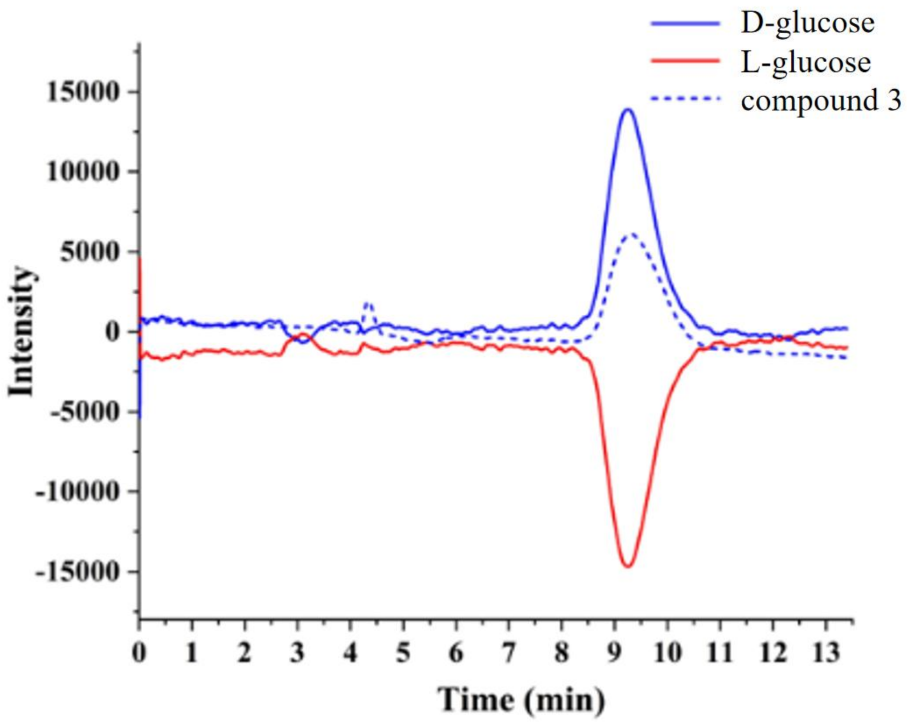


**Figure S17**. Determination of the glucose configuration of compound **3** by ORD (optical rotation detector) and HPLC.

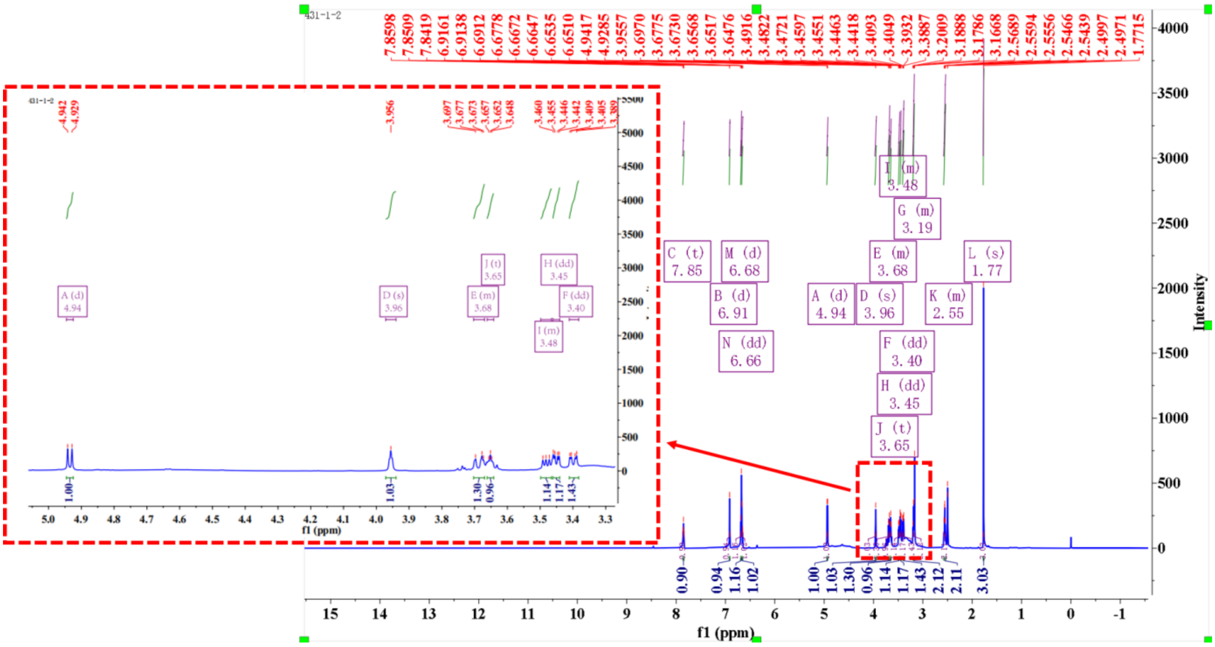


**Figure S18**. $^{1}H$ NMR spectrum (600 MHz) of compound **4** (DMSO-$d_6$).

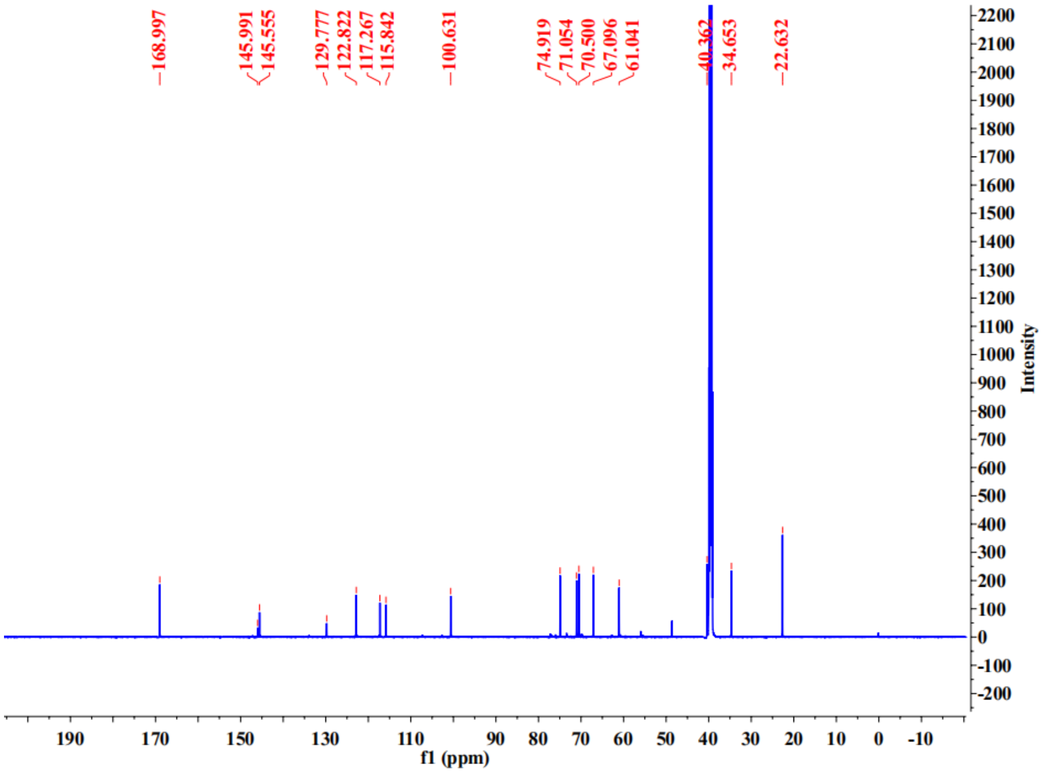


**Figure S19**. $^{13}C$ NMR spectrum (150 MHz) of compound **4** (DMSO-$d_6$).

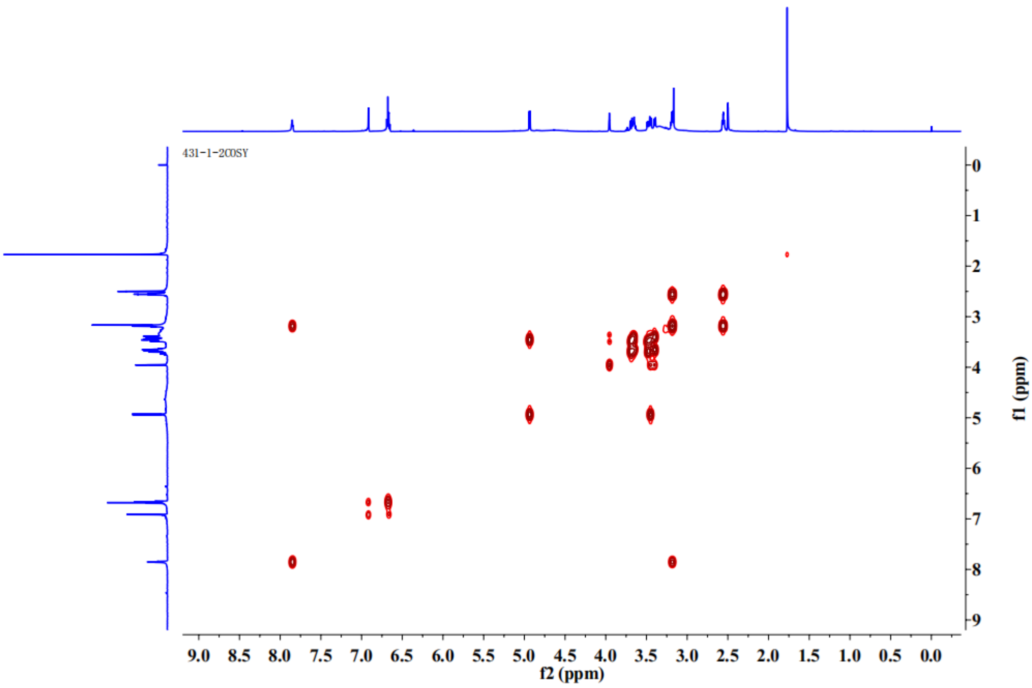


**Figure S20**. $^{1}H$-$^{1}H$ COSY spectrum of compound **4** (DMSO-*d*6).

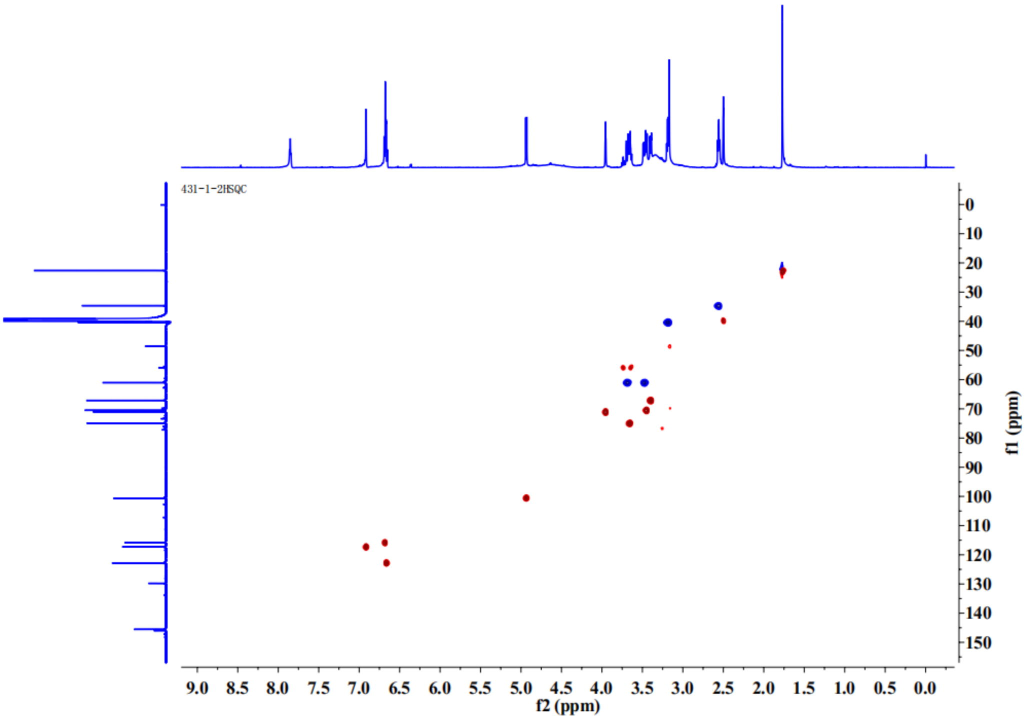


**Figure S21**. HSQC spectrum of compound **4** (DMSO-*d*6).

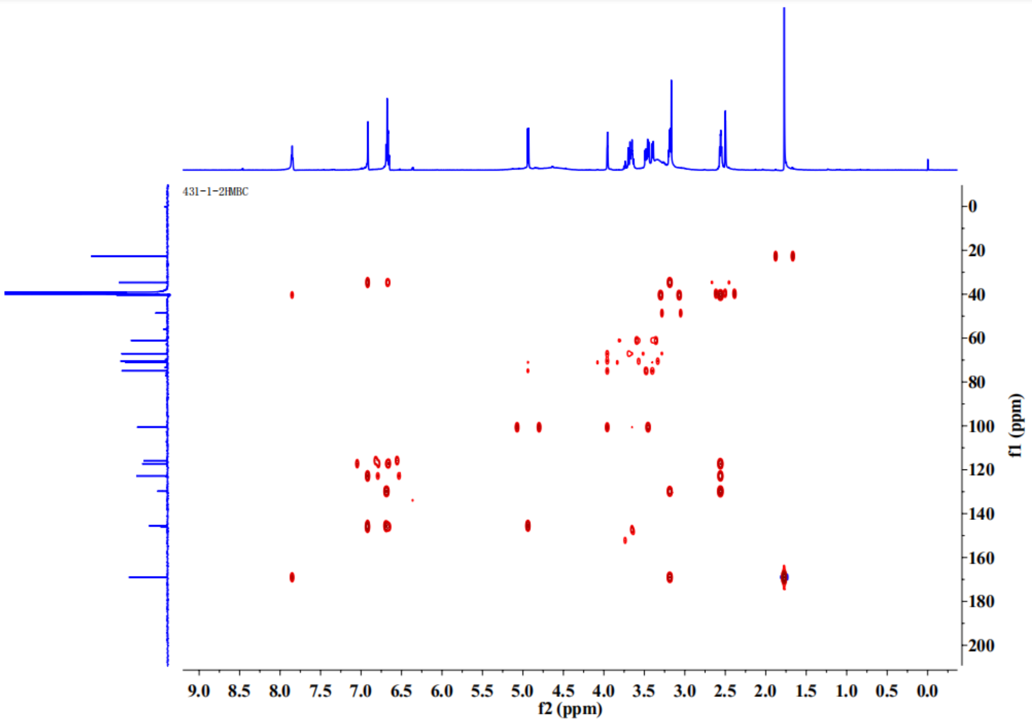


**Figure S22**. HMBC spectrum of compound **4** (DMSO-$d_6$).

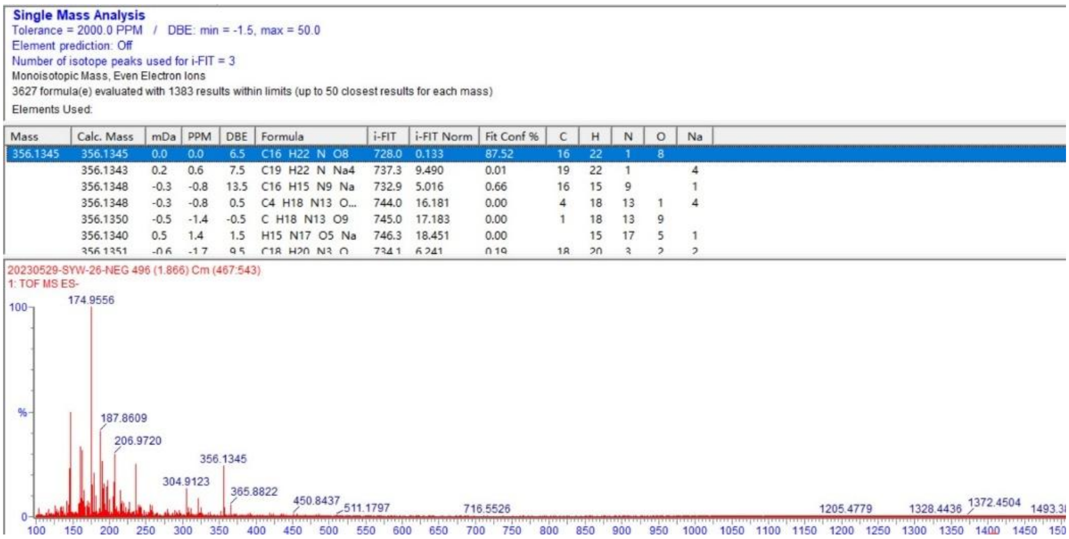

Single Mass Analysis
Tolerance = 2000.0 PPM / DBE: min = -1.5, max = 50.0
Element prediction: Off
Number of isotope peaks used for i-FIT = 3
Monoisotopic Mass, Even Electron Ions
3627 formula(e) evaluated with 1383 results within limits (up to 50 closest results for each mass)
Elements Used:

| Mass | Calc. Mass | mDa | PPM | DBE | Formula | i-FIT | i-FIT Norm | Fit Conf % | C | H | N | O | Na |
|---|---|---|---|---|---|---|---|---|---|---|---|---|---|
| 356.1345 | 356.1345 | 0.0 | 0.0 | 6.5 | C16 H22 N O8 | 728.0 | 0.133 | 87.52 | 16 | 22 | 1 | 8 | |
| | 356.1343 | 0.2 | 0.6 | 7.5 | C19 H22 N Na4 | 737.3 | 9.490 | 0.01 | 19 | 22 | 1 | | 4 |
| | 356.1348 | -0.3 | -0.8 | 13.5 | C16 H15 N9 Na | 732.9 | 5.016 | 0.66 | 16 | 15 | 9 | | 1 |
| | 356.1348 | -0.3 | -0.8 | 0.5 | C4 H18 N13 O... | 744.0 | 16.181 | 0.00 | 4 | 18 | 13 | 1 | 4 |
| | 356.1350 | -0.5 | -1.4 | -0.5 | C H18 N13 O9 | 745.0 | 17.183 | 0.00 | 1 | 18 | 13 | 9 | |
| | 356.1340 | 0.5 | 1.4 | 1.5 | H15 N17 O5 Na | 746.3 | 18.451 | 0.00 | | 15 | 17 | 5 | 1 |
| | 356.1351 | -0.6 | -1.7 | 9.5 | C18 H20 N3 O | 734.1 | 6.241 | 0.19 | 18 | 20 | 3 | 2 | 2 |



**Figure S23**. HRESITOFMS data of compound **4**.

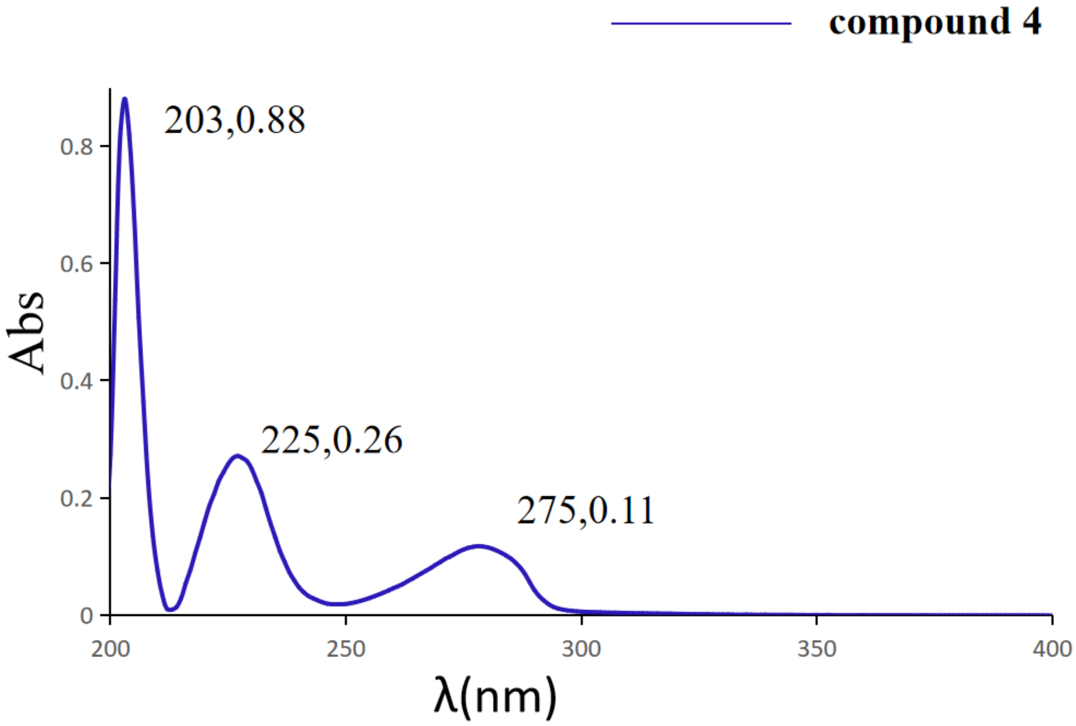


**Figure S24**. UV spectrum of compound **4**.

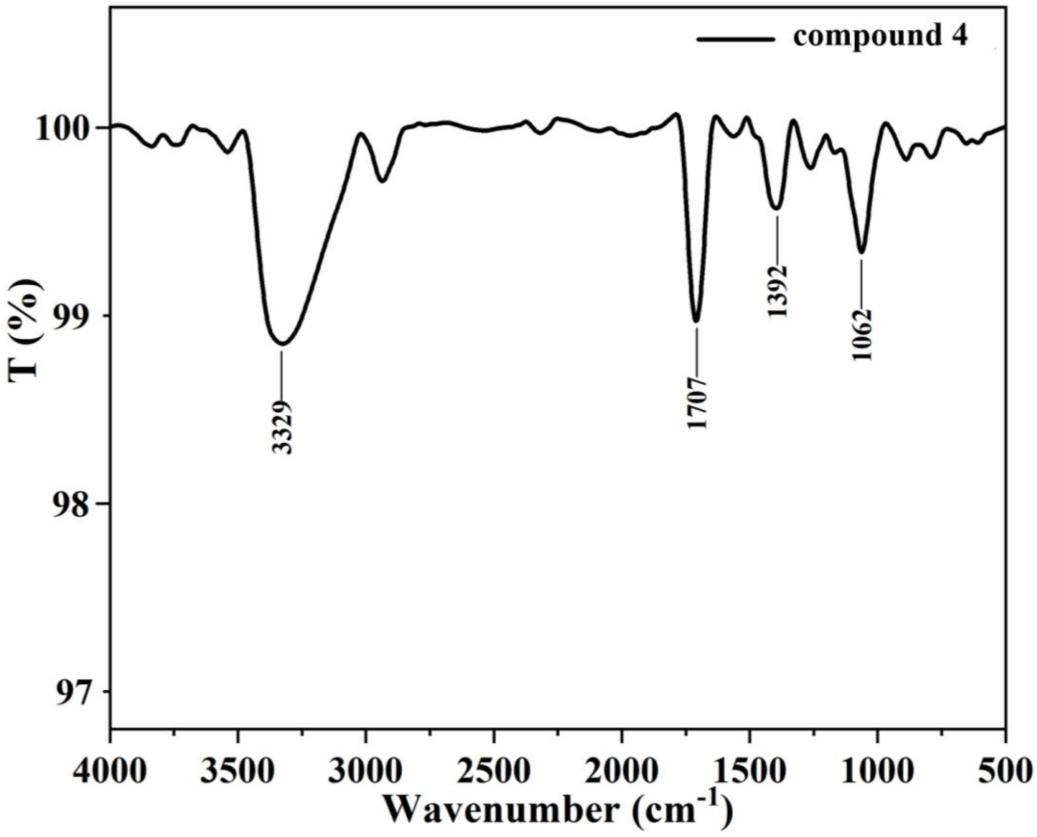


**Figure S25**. IR spectrum of compound **4**.

d6).

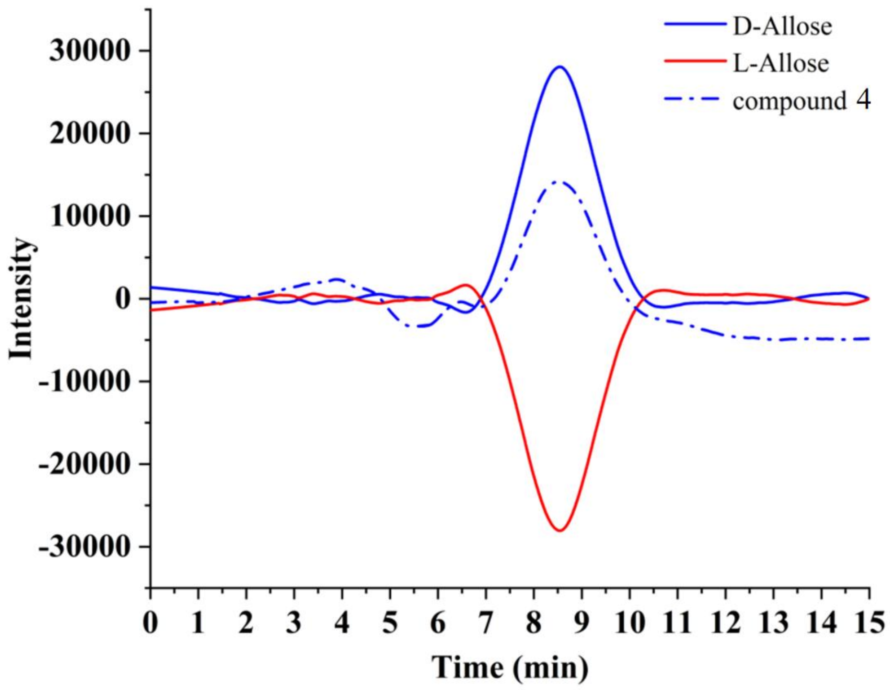


**Figure S26**. Determination of the allose configuration of compound **4** by ORD (optical rotation detector) and HPLC.

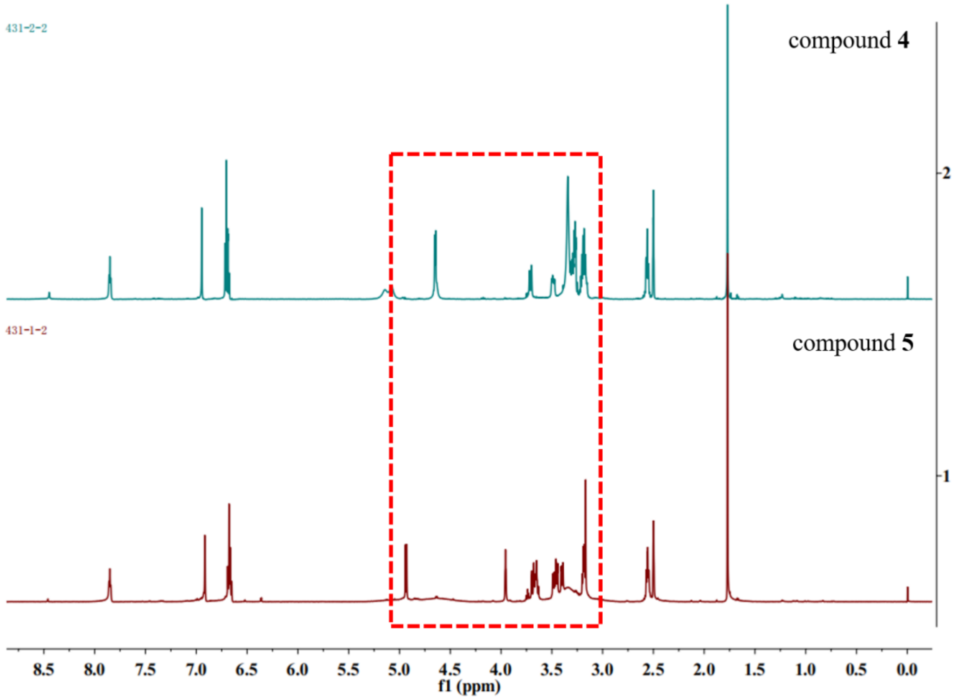


**Figure S27**. Comparison of the $^1$H NMR spectrum (600 MHz) of compounds **4** and **5** (DMSO-*d*6).
